# Pattern and Management of Mandibular Third Molar Impaction in Tirunelveli, India: A Cross-Sectional Study

**DOI:** 10.7759/cureus.113488

**Published:** 2026-07-28

**Authors:** S Sindhuja Devi, Kiran Kumar, Elakkiya S, Hamsavathy I, I Packiaraj, Sathyapriya Lakshmanan B

**Affiliations:** 1 Department of Oral and Maxillofacial Surgery, Bharath Institute of Higher Education and Research, Chennai, IND; 2 Department of Oral and Maxillofacial Surgery, Rajas Dental College and Hospital, Kavalkinaru, IND; 3 Department of Anatomy, Sree Balaji Dental College and Hospital, Chennai, IND

**Keywords:** classification, mandibular third molar, oral surgery, panoramic radiography, tooth impaction

## Abstract

Introduction

Mandibular third molar impaction is one of the most frequently encountered conditions in oral and maxillofacial surgery and is often associated with pain, infection, periodontal disease, and surgical complications. Understanding the pattern of impaction and its relationship with surgical management is essential for appropriate treatment planning and for minimizing postoperative morbidity. This study aimed to evaluate the pattern of mandibular third molar impaction and its association with surgical management, postoperative complications, age, and sex in patients attending a dental care institution.

Materials and methods

This institution-based cross-sectional study included 330 patients with impacted mandibular third molars, selected after screening 690 orthopantomograms (OPGs). Demographic information and radiographic characteristics were collected from clinical records and panoramic radiographs. The surgical approach and postoperative complications were also recorded. Categorical variables are expressed as n (%) and were analyzed using the chi-square test, while the independent samples t-test was used to compare continuous variables. Statistical significance was set at p < 0.05.

Results

Of 330 patients, 180 (54.5%) were men, and 150 (45.5%) were women. Mesioangular impaction was the most common pattern in 130 patients (39.4%), followed by vertical impaction in 80 patients (24.2%). Pell and Gregory Level B and Class II were the predominant radiographic classification. Bone removal with sectioning was the most commonly performed surgical procedure in 160 patients (48.5%), and postoperative complications were observed in 50 patients (15.2%). Significant associations were found between sex and impaction type (p = 0.005), impaction depth and surgical approach (p < 0.001), ramus relationship and postoperative complications (p < 0.001), and age and impaction pattern (p < 0.001).

Conclusion

Mesioangular impaction was the predominant mandibular third molar impaction pattern in the study population. Radiographic characteristics significantly influenced the choice of surgical management and the occurrence of postoperative complications. Comprehensive preoperative assessment using standardized classification systems facilitates the prediction of surgical difficulty and supports improved clinical decision-making and patient outcomes.

## Introduction

Mandibular third molars are the most frequently impacted teeth in permanent dentition, primarily due to inadequate retromolar space, delayed eruption, and variations in tooth angulation during development [1]. Impacted third molars may remain asymptomatic for years; however, they are often associated with pathological conditions such as pericoronitis, dental caries involving adjacent teeth, periodontal defects, root resorption, cystic lesions, and, less commonly, odontogenic tumors [2]. Third molar impaction is one of the most common indications for oral and maxillofacial surgical intervention, accounting for a substantial proportion of outpatient surgical procedures worldwide [2,3].

The pattern of mandibular third molar impaction varies among different populations, owing to differences in craniofacial morphology, genetic predisposition, dietary habits, and environmental factors. The classification systems proposed by Winter and Pell and Gregory remain the most widely accepted methods for assessing the angulation, depth, and relationship between impacted teeth and mandibular ramus [4]. These classifications are valuable not only for describing impaction patterns but also for predicting surgical difficulty, operative time, and the likelihood of intraoperative and postoperative complications. Accurate preoperative assessment facilitates appropriate treatment planning, patient counseling, and the selection of the most suitable surgical approach, thereby minimizing morbidity [5].

Although several epidemiological studies have evaluated the prevalence and characteristics of mandibular third molar impactions, regional variations continue to exist, and institution-specific data remain limited in many parts of India [6,7]. In particular, evidence from southern Tamil Nadu is scarce, despite potential differences in demographic characteristics, healthcare-seeking behavior, and clinical presentation that may influence impaction patterns and surgical management. Region-specific data are therefore needed to strengthen the evidence base for clinical decision-making in this population. Furthermore, understanding the relationship between impaction characteristics and surgical management can assist clinicians in anticipating procedural complexity and improving treatment outcomes in routine clinical practice. The primary objective of this study was to evaluate the pattern of mandibular third molar impaction among patients attending the Department of Oral and Maxillofacial Surgery at Rajas Dental College and Hospital, Tirunelveli, Tamil Nadu, India. The secondary objectives were to determine the association between impaction characteristics and the surgical management employed, assess the relationship between impaction characteristics and the occurrence of postoperative complications, and evaluate the association of age and sex with different patterns of mandibular third molar impaction.

## Materials and methods

Study design and study setting

This institution-based cross-sectional observational study was conducted in the Department of Oral and Maxillofacial Surgery at Rajas Dental College and Hospital, Tirunelveli, Tamil Nadu, India. The study was performed using orthopantomograms (OPGs) and the corresponding clinical records of patients who attended the Department of Oral and Maxillofacial Surgery between May 2024 and April 2025. The study protocol was reviewed and approved by the Institutional Ethical Committee of Rajas Dental College and Hospital before commencement (RDCH/PRL/IRB/D-2141/2025, dated April 18, 2025), and all procedures were conducted in accordance with the ethical principles of the Declaration of Helsinki. Patient confidentiality was strictly maintained by anonymizing all collected data.

Sample size estimation

The sample size was calculated using the formula for estimating a single population proportion:

\[ n = \frac{Z^{2} \times P \times (1 - P)}{d^{2}} \]

where n represents the required sample size, Z = 1.96 (corresponding to a 95% confidence level), P = 0.24 (24% expected prevalence of mandibular third molar impaction based on a previous Indian epidemiological study), and d = 0.05 (5% absolute precision) [6]. Therefore, the minimum required sample size was 280 participants. To compensate for incomplete records, poor-quality radiographs, and missing data, an additional 10% (28 participants) was added, resulting in a final target sample size of 308 participants. To further improve statistical precision, 330 eligible patients were ultimately included in the study.

Study population and sampling technique

**Figure 1:**
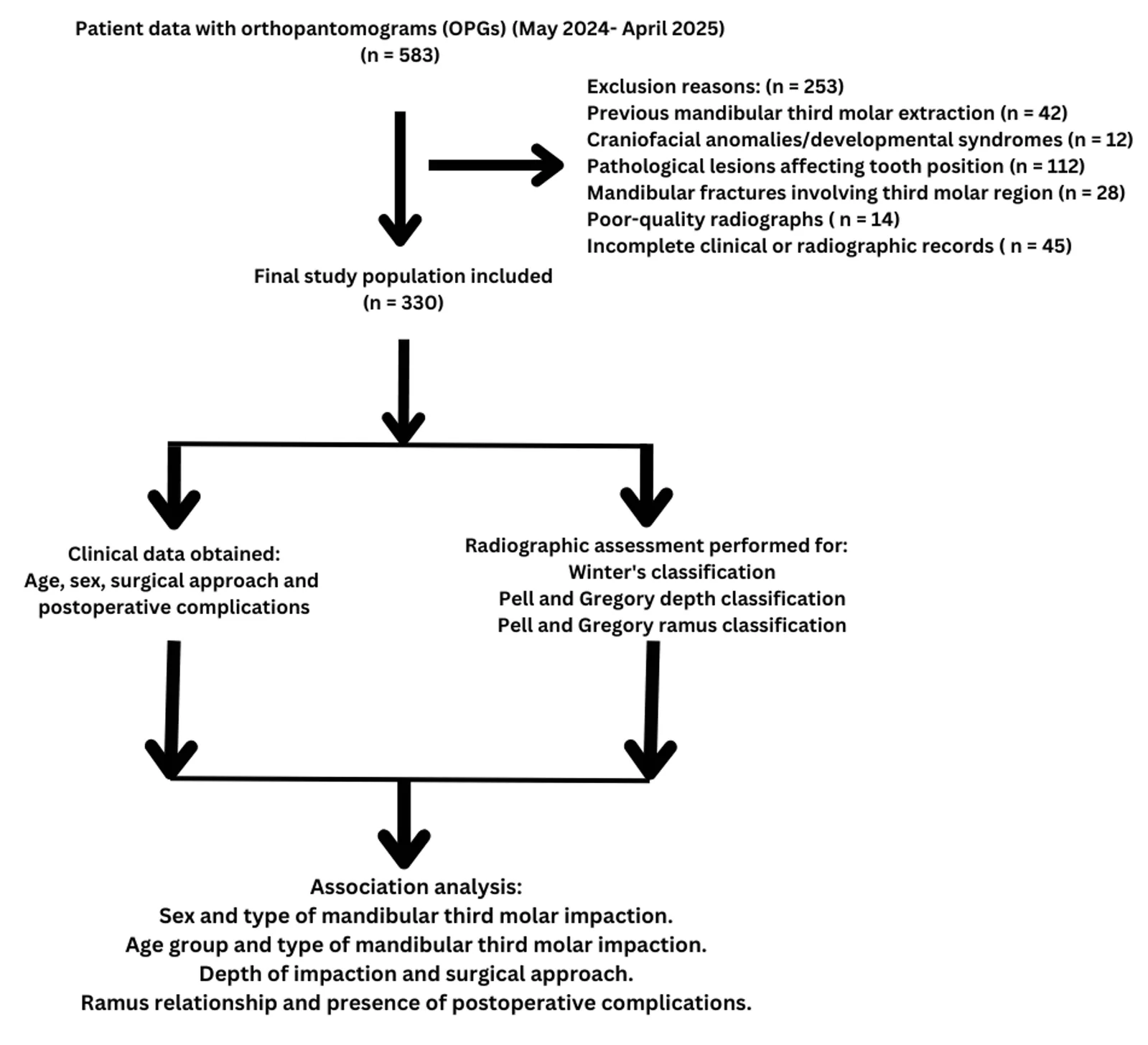
Study flow chart. Image created using Canva (Canva Pty Ltd, Sydney, Australia). No artificial intelligence-generated content was used in the preparation of this image.

A total of 690 OPGs were screened from the departmental archives. Following the eligibility assessment, 330 patients with at least one impacted mandibular third molar who fulfilled the selection criteria were included in the study. Consecutive sampling was employed until the desired sample size was achieved, thereby minimizing selection bias and ensuring adequate representation of patients attending the institution (Figure 1).

Eligibility criteria

Patients aged ≥18 years who presented with at least one impacted mandibular third molar on diagnostic-quality orthopantomogram were included in the study. Diagnostic-quality OPGs were defined as radiographs with adequate image sharpness, density, and contrast, free from significant positioning errors or motion artifacts, and providing the complete visualization of the mandibular third molars and adjacent anatomical structures. Only patients with complete demographic information, clinical records, and radiographs of acceptable diagnostic quality were eligible for inclusion. Patients with a previous mandibular third molar extraction, craniofacial anomalies, developmental syndromes affecting tooth eruption, pathological lesions causing the displacement of the third molars, mandibular fractures involving the third molar region, or incomplete clinical or radiographic records were excluded from the study.

Data collection procedure

Demographic and clinical information, including age, sex, radiographic findings, surgical procedure performed, and postoperative complications, was retrieved from patient case records. Each OPG was carefully evaluated by experienced oral and maxillofacial surgeons under standardized viewing conditions. All radiographs were independently assessed, and disagreements were resolved by consensus to ensure consistency in classification.

**Figure 2:**
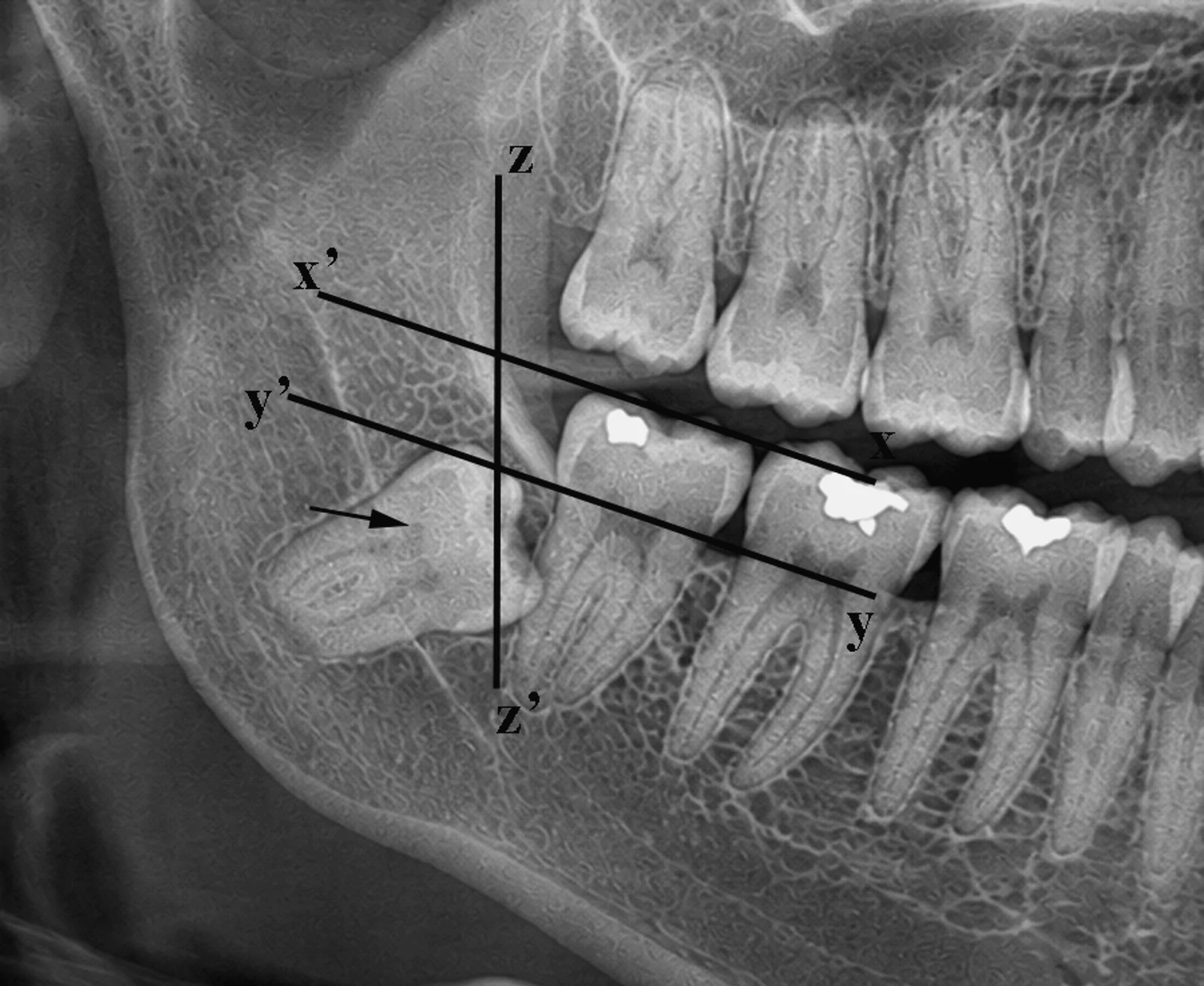
Representative panoramic radiograph demonstrating a mesioangular impacted mandibular third molar according to Winter's classification and classified as Pell and Gregory Level B and Class II. The x-x′ line represents the occlusal plane, the y-y′ line represents the reference line parallel to the occlusal plane used to assess the depth of impaction (Levels A-C), and the z-z′ line represents the anterior border of the mandibular ramus used to determine the ramus relationship (Classes I-III). The image is representative of the study sample.

Demographic and clinical data were systematically collected for each eligible participant from clinical records and corresponding orthopantomograms. The recorded variables included patient age, which was categorized into three groups (<25, 25-40, and >40 years), and sex. Radiographic assessment included the classification of mandibular third molar impaction according to Winter's classification (mesioangular, vertical, horizontal, and distoangular) [8], the depth of impaction using the Pell and Gregory classification (Levels A, B, and C), and relationship between the impacted tooth and mandibular ramus based on the Pell and Gregory ramus classification (Classes I, II, and III) [4]. Details of surgical management, including simple extraction, bone removal with tooth sectioning, or complex flap elevation with osteotomy, were obtained from operative records (Figure 2). Existing follow-up records were reviewed to identify postoperative complications, and patients were categorized according to the presence or absence of documented complications for statistical analysis.

Radiographic assessment

The angulation of impacted mandibular third molars was classified according to Winter's classification into mesioangular, vertical, horizontal, and distoangular impactions [8]. The depth of impaction relative to the occlusal plane was assessed using the Pell and Gregory depth classification, categorizing teeth as Level A, B, or C [4]. The relationship between the impacted tooth and anterior border of the mandibular ramus was determined using the Pell and Gregory ramus classification, which categorizes impactions into Class I, Class II, and Class III [4].

Surgical management assessment

The surgical treatment rendered for each impacted mandibular third molar was obtained from operative records and categorized into three groups: simple extraction, bone removal with tooth sectioning, and complex flap elevation with osteotomy. The selected surgical technique reflected the clinical difficulty anticipated from the preoperative radiographic assessment and the intraoperative findings documented by the operating surgeon.

Assessment of postoperative complications

Postoperative follow-up records were reviewed to determine the occurrence of complications after mandibular third molar surgery. Postoperative complications were defined as any adverse event requiring additional clinical management during the follow-up period, including alveolar osteitis (dry socket), surgical site infection requiring antibiotic therapy, persistent postoperative bleeding requiring intervention, trismus requiring additional treatment, inferior alveolar or lingual nerve sensory disturbance, and postoperative pain requiring an unscheduled clinical visit or additional analgesic prescription beyond the routine postoperative regimen. Patients were categorized according to the presence or absence of at least one postoperative complication. All patients underwent a routine postoperative review at approximately seven days after surgery, and only patients with documented follow-up at this visit were included in the analysis.

Calibration and reliability assessment

Prior to the commencement of data collection, the examiners underwent calibration for the radiographic assessment of mandibular third molar impactions using Winter's classification and the Pell and Gregory classification. A random sample of 30 OPGs (approximately 10% of the final sample) was independently evaluated on two separate occasions with a two-week interval to assess intra- and inter-examiner reliability. An experienced oral and maxillofacial surgeon independently assessed the radiographs to determine their reliability. The agreement for categorical variables was evaluated using Cohen's kappa (κ) coefficient. Kappa values of ≥0.80 were considered indicative of excellent agreement. The intra-examiner and inter-examiner kappa values were 0.91 and 0.88, respectively, demonstrating the excellent reproducibility of the radiographic assessments.

Statistical analysis

All collected data were verified for completeness before statistical analysis. Statistical analyses were performed using the IBM SPSS Statistics for Windows version 25.0 (IBM Corp., Armonk, NY). Categorical variables are summarized as frequencies and percentages (n, %), whereas continuous variables are expressed as mean and standard deviation after the assessment of data completeness. Associations between categorical variables, including sex, age group, Winter's classification, the Pell and Gregory classification, surgical approach, and postoperative complications, were evaluated using the chi-square test of independence. Effect sizes were calculated to determine the magnitude of the observed associations. Cramer's V was reported for chi-square analyses. All statistical tests were two-tailed, and statistical significance was set at p < 0.05.

## Results

A total of 690 OPGs were screened, of which 330 (47.8%) patients with impacted mandibular third molars fulfilled the eligibility criteria and were included in the final analysis. The study population comprised 180 men (54.5%) and 150 women (45.5%). Most participants were younger than 25 years of age. Mesioangular impaction was the most frequently observed pattern, followed by vertical impaction, whereas Pell and Gregory Level B and Class II were the predominant depth and ramus classifications, respectively. Bone removal with sectioning was the most commonly performed surgical procedure, and postoperative complications were observed in 50 patients (15.2%). The mean follow-up time was 35.5 ± 12.5 days (Table 1).

**Table 1:** Demographic and clinical characteristics of the study population (n = 330). Data are presented in the form of mean and standard deviation (SD), median, interquartile range (IQR), and frequency (n) and percentage (%).

Variable	Category	n (%)
Gender	Male	180 (54.5%)
	Female	150 (45.5%)
Age group	<25 years	150 (45.5%)
	25-40 years	120 (36.4%)
	>40 years	60 (18.2%)
Winter's classification	Mesioangular	130 (39.4%)
	Vertical	80 (24.2%)
	Horizontal	70 (21.2%)
	Distoangular	50 (15.2%)
Pell-Gregory depth (level)	Level A (high)	100 (30.3%)
	Level B (medium)	150 (45.5%)
	Level C (low/deep)	80 (24.2%)
Pell-Gregory ramus (class)	Class I (space present)	110 (33.3%)
	Class II (moderate space)	140 (42.4%)
	Class III (no space)	80 (24.3%)
Surgical approach	Simple extraction	80 (24.2%)
	Bone removal + sectioning	160 (48.5%)
	Complex flap + osteotomy	90 (27.3%)
Postoperative complications	Present	50 (15.2%)
	Absent	280 (84.8%)
Follow-up period, days	Mean ± SD	35.5 ± 12.5
	Median (IQR)	32.0 (23.6-40.4)

A statistically significant association was observed between sex and Winter's classification of mandibular third molar impaction (p = 0.005). Mesioangular impaction was more frequently observed among men, whereas horizontal and distoangular impactions were relatively more common among women (Table 2).

**Table 2:** Association between gender and the type of mandibular third molar impaction according to Winter's classification. Data are presented as n (%), associations were analyzed using the Pearson chi-square test, effect size was estimated using Cramer's V, and p < 0.05 was considered statistically significant. *Statistically significant. χ², chi-square statistic; df, degrees of freedom

Sex	Mesioangular	Vertical	Horizontal	Distoangular	Total	χ² (df)	P‑value	Cramer's V
Male	80 (44.4%)	50 (27.8%)	30 (16.7%)	20 (11.1%)	180 (100%)	12.80 (3)	0.005*	0.197
Female	50 (33.3%)	30 (20.0%)	40 (26.7%)	30 (20.0%)	150 (100%)			
Total	130 (39.4%)	80 (24.2%)	70 (21.2%)	50 (15.2%)	330 (100%)			

The depth of impaction was significantly associated with the surgical approach employed (p < 0.001). Level A impactions were predominantly managed by simple extraction (50.0%), whereas Level B impactions most frequently required bone removal with sectioning (53.3%). Although the majority of Level C impactions required bone removal with sectioning (50.0%) or complex flap elevation with osteotomy (37.5%), a small proportion (12.5%) was managed by simple extraction. Overall, deeper impactions were more likely to require more invasive surgical procedures; however, the choice of surgical approach depended on the overall clinical and radiographic assessment rather than the depth of impaction alone (Table 3).

**Table 3:** Association between the Pell-Gregory depth of impaction and surgical approach. Data are presented as n (%), associations were analyzed using the Pearson chi-square test, effect size was estimated using Cramer's V, and p < 0.05 was considered statistically significant. *Statistically significant. χ², chi-square statistic, df, degrees of freedom

Depth level	Simple extraction	Bone removal + sectioning	Complex flap + osteotomy	Total	χ² (df)	P‑value	Cramer's V
Level A (high)	50 (50.0%)	40 (40.0%)	10 (10.0%)	100 (100%)	57.77 (4)	<0.001*	0.296
Level B (medium)	20 (13.3%)	80 (53.3%)	50 (33.3%)	150 (100%)			
Level C (low/deep)	10 (12.5%)	40 (50.0%)	30 (37.5%)	80 (100%)			
Total	80 (24.2%)	160 (48.5%)	90 (27.3%)	330 (100%)			

**Figure 3:**
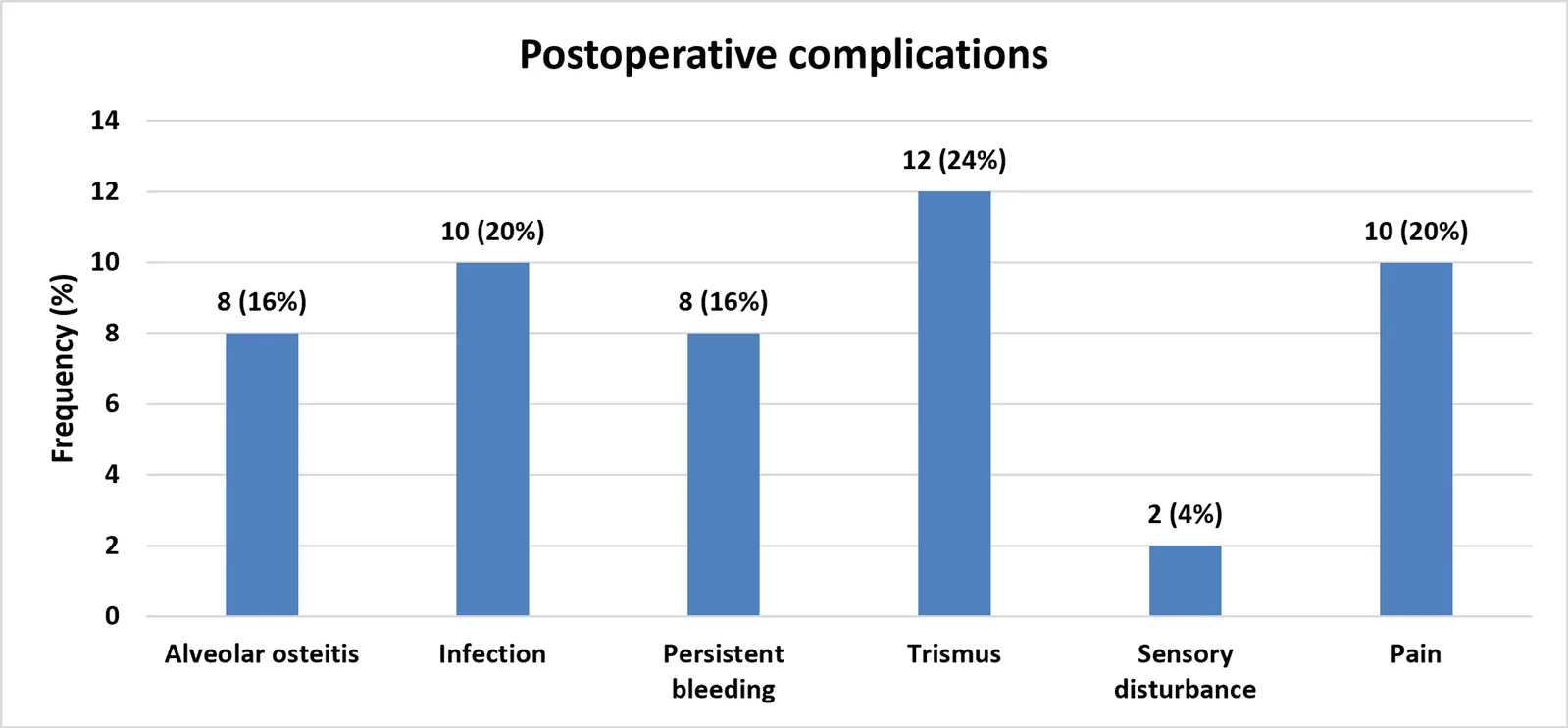
Postoperative complications (n = 50) during the follow-up period.

The most frequent postoperative complication following impacted tooth surgery was trismus, affecting 12 (24%) of patients. Infection and pain each occurred in 10 (20%) cases, while alveolar osteitis and persistent bleeding were observed in 16% each. Sensory disturbance was the least common, reported in only two (4%) patients, indicating a generally favorable neurological outcome (Figure 3).

A significant relationship was also identified between the Pell and Gregory ramus classification and the occurrence of postoperative complications (p < 0.001). Patients with Class III impactions exhibited the highest proportion of complications compared with those with Class I or Class II impactions (Table 4).

**Table 4:** Association between the Pell-Gregory ramus relationship and postoperative complications. Data are presented as n (%), associations were analyzed using the Pearson chi-square test, effect size was estimated using Cramer's V, and p < 0.05 was considered statistically significant. *Statistically significant. χ², chi-square statistic, df, degrees of freedom

Ramus class	Postoperative complications present	Postoperative complications absent	Total	χ² (df)	P‑value	Cramer's V
Class I (space present)	10 (9.1%)	100 (90.9%)	110 (100%)	21.50 (2)	<0.001*	0.255
Class II (moderate space)	15 (10.7%)	125 (89.3%)	140 (100%)			
Class III (no space)	25 (31.3%)	55 (68.7%)	80 (100%)			
Total	50 (15.2%)	280 (84.8%)	330 (100%)			

The distribution of mandibular third molar impaction patterns varied significantly across the different age groups (p < 0.001). Mesioangular impaction was predominant among younger patients, whereas distoangular impaction was relatively more frequent in older individuals, suggesting age-related differences in the impaction pattern (Table 5).

**Table 5:** Association between age group and the type of mandibular third molar impaction according to Winter's classification. Data are presented as n (%), associations were analyzed using the Pearson chi-square test, effect size was estimated using Cramer's V, and p < 0.05 was considered statistically significant. *Statistically significant. χ², chi-square statistic, df, degrees of freedom

Age group	Mesioangular	Vertical	Horizontal	Distoangular	Total	χ² (df)	P‑value	Cramer's V
<25 years	70 (46.7%)	40 (26.7%)	30 (20.0%)	10 (6.7%)	150 (100%)	27.28 (6)	<0.001*	0.203
25-40 years	40 (33.3%)	30 (25.0%)	30 (25.0%)	20 (16.7%)	120 (100%)			
>40 years	20 (33.3%)	10 (16.7%)	10 (16.7%)	20 (33.3%)	60 (100%)			
Total	130 (39.4%)	80 (24.2%)	70 (21.2%)	50 (15.2%)	330 (100%)			

## Discussion

This study evaluated the pattern of mandibular third molar impaction and its associations with surgical management and postoperative outcomes in patients attending a tertiary dental care institution. Mesioangular impaction was the most frequently observed angulation, followed by vertical and horizontal impactions. In addition, Pell and Gregory Level B and Class II represented the most common radiographic classifications. Significant associations were observed between sex and impaction type, impaction depth and surgical approach, ramus relationship and postoperative complications, and age group and impaction pattern. These findings highlight the clinical relevance of comprehensive radiographic assessment using standardized classification systems to support treatment planning and clinical decision-making.

Mesioangular impaction was the predominant pattern observed in the present study, accounting for approximately 40% of all impacted mandibular third molars. This finding is consistent with previous studies by Quek et al. [9], Hashemipour et al. [10], Passi et al. [6], and Hattab et al. [11], which similarly identified mesioangular impaction as the most common angulation across different populations. The predominance of this impaction pattern has been attributed to the mesial inclination of the developing mandibular third molar and inadequate retromolar space during eruption. The consistency of these findings across diverse populations suggests that developmental and anatomical factors contribute substantially to the distribution of mandibular third molar impactions.

The present study also demonstrated a significant association between sex and impaction pattern, with mesioangular impactions occurring more frequently among men, whereas horizontal and distoangular impactions were relatively more common among women. Comparable observations have been reported by Passi et al. [6], Alsaegh et al. [12], and Alsharif and Alsharif [13], who proposed that differences in mandibular growth, skeletal maturation, and eruption timing may contribute to variations in impaction patterns between sexes. Nevertheless, other investigators have reported no significant sex-related differences, indicating that these associations may vary among different populations and should be interpreted cautiously [14,15].

A significant association was observed between the depth of impaction and the surgical approach employed. Level A impactions were more commonly managed by simple extraction, whereas Level B and Level C impactions more frequently required bone removal with tooth sectioning or osteotomy. These findings are consistent with previous reports by Pell and Gregory [4], de Santana-Santos et al. [16], and Glodha et al. [3], which described an association between greater impaction depth and the need for more extensive surgical procedures. Deeper impactions often require increased bone removal and broader surgical access because of their anatomical position. Accordingly, the radiographic assessment of impaction depth provides useful information that may assist clinicians in selecting an appropriate surgical approach.

The present study further demonstrated a significant association between the Pell and Gregory ramus classification and postoperative complications. Patients with Class III impactions showed a higher proportion of postoperative complications than those with Class I and Class II impactions. Similar findings have been reported by Jerjes et al. [17], Susarla and Dodson [18], and Glodha et al. [3], who observed that deeply positioned third molars and reduced retromolar space were more frequently associated with technically demanding surgical procedures and postoperative adverse events. Although the present study identified an association between ramus classification and postoperative complications, the cross-sectional design does not permit conclusions regarding causality.

Age-related differences in impaction patterns were also identified. Mesioangular impactions were more frequently observed among younger patients, whereas distoangular impactions were relatively more common in older individuals. Similar trends have been described by Hashemipour et al. [10] and Passi et al. [6]. However, the observed association should not be interpreted as evidence that age influences tooth angulation. Instead, it may reflect differences in the timing of patient presentation, referral patterns, healthcare-seeking behavior, or clinical indications for radiographic examination across age groups. Consequently, these findings should be interpreted as describing the distribution of impaction patterns within the study population rather than indicating a causal relationship.

The present study contributes additional data on mandibular third molar impaction patterns from southern Tamil Nadu, a region for which published evidence remains limited. In addition to describing the distribution of impaction patterns, the study examined their associations with surgical management and postoperative complications using standardized radiographic classifications. The observed associations between impaction depth and surgical approach and between ramus relationship and postoperative complications were consistent with previous reports and support the continued clinical use of Winter's classification and the Pell and Gregory classification as standardized methods for radiographic assessment during preoperative evaluation. These findings may assist clinicians in treatment planning, patient counseling, and communication regarding the anticipated surgical approach while providing region-specific data for comparison with other populations.

Clinical implications

The findings of the present study highlight the importance of routine radiographic evaluation using standardized classification systems before mandibular third molar surgery. Winter's classification, together with the Pell and Gregory classification, provides a standardized framework for describing the angulation, depth, and ramus relationship of impacted mandibular third molars during preoperative assessment. These radiographic findings can assist clinicians in treatment planning, patient counseling, and selecting an appropriate surgical approach based on the individual clinical presentation. They also facilitate communication among clinicians and contribute to the more consistent documentation of impacted mandibular third molars. The region-specific findings of the present study may further support clinical decision-making and serve as a reference for future studies evaluating mandibular third molar impactions in similar populations.

Strengths

The strengths of the present study include its relatively large sample size, the use of well-established radiographic classification systems (Winter's classification and the Pell and Gregory classification), and the application of appropriate statistical analyses to evaluate the associations between impaction characteristics, surgical management, and postoperative complications. These methodological strengths enhance the reliability and clinical relevance of the findings.

Limitations

The present study has certain limitations. Because this was a single-center cross-sectional study, the findings may not be generalizable to other populations with different demographic characteristics. The use of retrospective clinical records restricted the evaluation of variables that had been routinely documented. Other factors influencing surgical difficulty, including root morphology, proximity to the inferior alveolar canal, the duration of surgery, and surgeon experience, were not assessed. In addition, postoperative complications were evaluated qualitatively rather than according to their severity or duration. Furthermore, the Pederson Difficulty Index was not used to quantify surgical difficulty. Although Winter's classification and the Pell and Gregory classification provide standardized radiographic assessment of impaction characteristics, incorporating a validated surgical difficulty index in future studies may improve the prediction of operative complexity and facilitate comparison with other published reports. Future multicenter prospective studies incorporating cone beam computed tomography, comprehensive clinical variables, and standardized surgical difficulty indices are recommended to provide a more comprehensive understanding of the factors influencing mandibular third molar impaction and surgical outcomes.

## Conclusions

The present study demonstrated that mesioangular impaction was the most common pattern of mandibular third molar impaction, with Pell and Gregory Level B and Class II being the predominant radiographic classifications. Significant associations were observed between impaction characteristics, surgical approach, postoperative complications, age, and sex. These findings highlight the importance of comprehensive preoperative radiographic assessment using established classification systems to support treatment planning, patient counseling, and clinical decision-making in the management of impacted mandibular third molars.
